# Genome-Wide DNA Methylation Profiling in Cultured Eutopic and Ectopic Endometrial Stromal Cells

**DOI:** 10.1371/journal.pone.0083612

**Published:** 2014-01-23

**Authors:** Yoshiaki Yamagata, Koichiro Nishino, Eiichi Takaki, Shun Sato, Ryo Maekawa, Akira Nakai, Norihiro Sugino

**Affiliations:** 1 Department of Obstetrics and Gynecology, Yamaguchi University Graduate School of Medicine, Ube, Japan; 2 Laboratory of Veterinary Biochemistry and Molecular Biology, Faculty of Agriculture, University of Miyazaki, Miyazaki, Japan; 3 Department of Biochemistry and Molecular Biology, Yamaguchi University Graduate School of Medicine, Ube, Japan; University of Bonn, Institut of Experimental Hematology and Transfusion Medicine, Germany

## Abstract

The objective of this study was to characterize the genome-wide DNA methylation profiles of isolated endometrial stromal cells obtained from eutopic endometria with (euESCa) and without endometriosis (euESCb) and ovarian endometrial cysts (choESC). Three samples were analyzed in each group. The infinium methylation array identified more hypermethylated and hypomethylated CpGs in choESC than in euESCa, and only a few genes were methylated differently in euESCa and euESCb. A functional analysis revealed that signal transduction, developmental processes, immunity, etc. were different in choESC and euESCa. A clustering analysis and a principal component analysis performed based on the methylation levels segregated choESC from euESC, while euESCa and euESCb were identical. A transcriptome analysis was then conducted and the results were compared with those of the DNA methylation analysis. Interestingly, the hierarchical clustering and principal component analyses showed that choESC were segregated from euESCa and euESCb in the DNA methylation analysis, while no segregation was recognized in the transcriptome analysis. The mRNA expression levels of the epigenetic modification enzymes, including DNA methyltransferases, obtained from the specimens were not significantly different between the groups. Some of the differentially methylated and/or expressed genes (NR5A1, STAR, STRA6 and HSD17B2), which are related with steroidogenesis, were validated by independent methods in a larger number of samples. Our findings indicate that different DNA methylation profiles exist in ectopic ESC, highlighting the benefits of genome wide DNA methylation analyses over transcriptome analyses in clarifying the development and characterization of endometriosis.

## Introduction

Endometriosis, which affects approximate 10% of reproductive-aged females, is defined as the ectopic presence of endometrial-like tissue in the ovaries, retroperitoneum, rectum, etc. It causes chronic pelvic pain, especially during menstruation and intercourse, and infertility. The rate of endometriosis has been increasing and the effects on women's health and social lives are not negligible. The elucidation of the pathogenesis of endometriosis is not complete and a better understanding of the molecular mechanisms underlying the aberrant gene expression observed in endometriosis would be of great importance in preventing and establishing effective treatments for this benign yet bothersome disease.

Endometriosis develops in response to sex steroid hormone exposure, such as estrogen exposure, after menarche. Although there is no definitive consensus regarding the histologic origin of endometriosis, several mechanisms have been proposed, including endometrial tissue transplantation, coelomic metaplasia, endometrial tissue metastasis via veins or lymphatic vessels, stem cell differentiation into endometriotic tissue or a combination of these mechanisms. Only one of these theories is unable to completely explain all of the phenomena related to the origin of endometriosis observed to date. Sampson has proposed a hypothesis that fragments of menstrual endometrial tissue pass backwards through the fallopian tubes and are implanted onto the surfaces of pelvic organs such as the ovaries and retroperitoneum [1]. While most females experience reverse flow of the menstrual endometrium into the pelvic cavity, Sampson's implantation hypothesis is still widely accepted and his observations imply that aberrant host immune responses may be involved in the initiation and progression of endometriosis.

To understand the molecular features of endometriosis, functional analyses of key genes and robust transcriptome analyses have been conducted. These studies demonstrate that the presence of endometriotic lesions indicates the occurrence of abnormal functions, such as abnormal estrogen production, progesterone resistance, inflammation, abnormal immune responses, etc., compared to that observed in eutopic endometria. At the molecular level, the levels of nuclear receptors, such as steroidgenic factor 1 [2], [3], estrogen receptor β [4] and progesterone receptors [5], are different in endometriotic tissue and eutopic endometria. Moreover, endometriotic tissue expresses different signaling pathways compared to eutopic endometria [6]. In spite of these findings, we still face complexity regarding comprehension of the underlying mechanisms of endometriosis.

Epigenetic modification mechanisms are undoubtedly recognized as key players in transcriptional regulation, and numerous reports indicate that aberrant modifications may be associated with the onset of various kinds of human diseases. DNA methylation is one of the most important and most studied chemical modifications. Accumulating data suggests that aberrant DNA methylation status may be associated with the molecular features of endometriosis. Previous reports have shown that abnormal promoter methylation occurs in certain genes, such as progesterone receptor B [5], E-cadherin [7], HOXA10 [8], estrogen receptor β [4], steroidgenic factor 1 [2], [3] and aromatase [9], [10]. These results suggest that epigenetic abnormalities are important in the pathogenesis and development of endometriosis; however, these results do not elucidate to what extent DNA methylation status contributes to endometriosis at the genome-wide level. Genome-wide DNA methylation analysis is an emerging field of research and several methods are available to detect the levels of methylation. We performed the analysis using a microarray-based method that is able to measure the precise levels of DNA methylation. This analysis was accompanied by a transcriptome analysis of isolated and cultured endometrial stromal cells (ESC) obtained from eutopic endometria and ovarian endometrial cysts.

## Materials and Methods

This study was reviewed and approved by the Institutional Review Board of Yamaguchi University Graduate School of Medicine. Written informed consent was obtained from the participants before the collection of any samples, and the specimens were irreversibly de-identified. All experiments handling human cells and tissues were performed in accordance with Tenets of the Declaration of Helsinki.

### Cell culture

Eutopic endometria with and without endometriosis were collected. Endometrial chocolate cysts were also collected. Clinical stages of endometriosis using revised American Fertility Society classification were moderate or severe. ESC were isolated as previously reported with a slight modification [11], [12]. The endometrial tissues and chocolate cysts were washed with phenol red-free Dulbecco's modified Eagle's medium (DMEM) (Invitrogen, Paisley, UK) containing Glutamax (Invitrogen), 50 µg/ml of streptomycin (Invitrogen) and 50 IU/ml of penicillin (Invitrogen) and then minced into small pieces measuring <1 mm^3^. After that, enzymatic digestion of the minced tissues with 0.2% collagenase (Sigma, St. Louis, MO, USA) was performed in a shaking incubator for two hours at 37°C, and then the ESC were separated using filtration through a 70 µm nylon mesh. The filtrates were washed three times. The ESC were seeded in 75 cm^2^ tissue culture flasks and grown until confluence in phenol red-free DMEM containing Glutamax, antibiotics and 10% dextran-coated charcoal-stripped fetal calf serum (Biological Industries, Kibbutz Beit Haemek, Israel) at 37°C in 95% air and 5% CO2. The homogeneity of the isolated ESC preparation was 98%, which was verified by immunocytochemistry using an antibody against vimentin, a specific marker of stromal cells. ESC were similarly cultured after isolation in all experiments of this study. The ESC were collected for analysis five to six days after isolation. If necessary, the cells were subcultured into another 75 cm^2^ tissue culture flask. Only one successive subculture was done before cell harvest. The ESC from eutopic endometria without endometriosis, the ESC from eutopic endometria with endometriosis and the ESC from chocolate cysts were defined as euESCa, euESCb and choESC, respectively.

### DNA and RNA isolation

DNA and RNA was isolated from 2–4×10^6^ cells using the RNeasy and DNeasy, respectively, from QIAGEN (Valencia, CA, USA) in accordance with the manufacturer's instructions.

### Illumina Infinium HumanMethylation27 BeadChip assay

The DNA methylation analysis was performed using the Illumina infinium assay with the HumanMethylation27 BeadChip (Illumina, San Diego, CA, USA), which interrogates a total of 27,578 CpG sites primarily spread across the proximal promoter regions of transcription start sites of 14,475 consensus coding sequences. Methylated and unmethylated signals were used to compute β-values, which are quantitative scores of the DNA methylation levels, ranging from “0,” thus indicating completely unmethylated, to “1,” indicating completely methylated. The BeadChip was scanned on a BeadArray Reader (Illumina) according to the manufacturer's instructions. CpG sites with “detection *p* values”>0.05 (computed from the background based on negative controls) were eliminated from further analysis, leaving 27,527 CpGs valid for use with the nine samples tested. The microarray data of DNA methylation is available at the Gene Expression Omnibus Web site (http://www.ncbi.nlm.nih.gov/geo/) under accession No. GSE47361.

### Analysis of DNA methylation data

The methylation data were analyzed using the following tools. Clustering was performed using an NIA array that classifies DNA methylation data by similarity and a principal component analysis (PCA) that detects major components in data variability [13]. To systematically annotate and predict the biological processes and pathways of differentially methylated genes in the euESCa and choESC, we used the following annotation sources: DAVID bioinformatics resources v6.7, PANTHER Classification System and the Kyoto Encyclopedia of Genes and Genomes (KEGG) [14], [15]. DAVID was used to determine whether the functional annotation of differentially methylated genes was enriched for specific Gene Ontology (GO) terms and KEGG pathways. Statistical significance was assessed with a modified Fisher's exact test. *p*<0.05 (after Benjamini and Hochberg correction for multiple testing) was considered significant enrichment.

### Real-time PCR array

Reverse transcription of RNA into cDNA was performed using the RT^2^ First Strand Kit (QIAGEN) in accordance with the instruction manual. In this study, the RT^2^ *Profiler*™ PCR Array, “Human Epigenetic Chromatin Modification Enzymes,” (QIAGEN) covering 84 genes encoding either known or predicted to modify genomic DNA and histones was applied. Real-time PCR was performed using the RT^2^ SYBR Green Master Mix (QIAGEN) according to the manufacturer's protocol under the following cycler conditions: 95°C: 10 min; 40 cycles (95°C: 15 s; 60°C: 60 s) using the Applied Biosystems 7700 Real-time PCR cycler (Applied Biosystems, Darmstadt, Germany). The relative quantity of cDNA was calculated with the ΔΔC_t_ method using five normalization genes: β-2-microglobulin, hypoxanthine phosphoribosyltransferase 1, ribosomal protein L13a, glyceraldehydes-3-phosphate dehydrogenase and β actin. A significant change in gene expression among the analyzed cell groups was defined as at least a 2-fold up- or downregulation of genes with *p*<0.01.

### Transcriptome analysis

To evaluate RNA integrity, a microfluidic analysis was performed using the Agilent 2100 Bioanalyzer with the RNA6000 nano kit (Agilent Technologies, Palo Alto, CA, USA). For the microarray analysis, we used only RNA samples whose RNA integrity number (RIN) was greater than 8.5. Gene expression was analyzed using a GeneChip® Human Gene 1.0 ST Array (Affymetrix, Santa Clara, CA, USA) containing 764,885 probes (and supporting 28,869 genes). Target cDNA was prepared from 200 ng of total RNA with the Ambion® WT Expression kit (Ambion, Austin, TX, USA) and the Affimetrix® GeneChip® WT Terminal Labeling kit (Affymetrix). Hybridization to the microarrays, washing, staining and scanning were performed using the GeneChip® system (Affymetrix) composed of the Scanner 3000 7G Workstation Fluidics 450 and the Hybridization Oven 645. The scanned image data were processed using the Affymetrix® Expression Console™ Version 1.1. Fold-change for each gene was evaluated using a Gene Expression Analysis with the Partek® Genomics Suite 6.5 software program (Partech, Münster, Germany). Genes expressing greater than 2-fold or less than 0.5 were recognized as significantly different. The microarray data of mRNA expression is available at the Gene Expression Omnibus Web site (http://www.ncbi.nlm.nih.gov/geo/) under accession No. GSE47361.

### Real-time reverse transcription (RT)-PCR

In the differentially methylated and expressed genes in choESC compared with euESCa, which were extracted by the Infinium 27K BeadsChiP and transcriptome analysis, we focused on the steroidogenesis-related genes, NR5A1, which encodes steroidogenic factor-1 (SF-1), STAR, which encodes steroidogenic acute regulatory protein, STRA6, which encodes stimulated by retinoic acid 6, and HSD17B2, which encodes 17β-hydroxysteroid dehydrogenase-2. To validate the results of the transcriptome analysis, real-time RT-PCR was conducted on these genes using a larger number of samples, including isolated euESCa (n = 7) and choESC (n = 5), endometrial tissues (n = 17), and chocolate cysts (n = 6).

RT reactions were performed with PrimeScript RT Master Mix (TAKARA, Ohtsu, Japan) according to the manufacturer's protocol. Briefly, 0.5 µg of total RNA was incubated with 4 µl of 5× PrimeScript RT Master Mix in 20 µl of reaction mixture at 37°C for 15 min, and the reverse transcriptase was inactivated by heating the samples at 85°C for 5 sec. The complementary DNA (cDNA) was immediately used for PCR. All PCR reactions were performed using SYBR Premix Ex Taq (TAKARA) and a LightCycler instrument (Roche Applied Science, Basel, Switzerland). Briefly, 2 µl aliquots containing cDNA were amplified in a total volume of 20 µl containing 4 µl of 5× SYBR PreMix Ex Taq and 0.2 µM of each primer. As an internal control for the RT-PCR, TATA box-binding protein (TBP) cDNA was also amplified. The following primers were used: NR5A1 (forward, 5′- GGAGTTTGTCTGCCTCAAGTTCA-3′, reverse, 5′- CGTCTTTCACCAGGATGTGGTT-3′) (80 bp); STAR (forward, 5′- GAACCCCAATGTCAAGGAGA-3′, reverse, 5′- CAGCCAGCTCGTGAGTAATG-3′) (74 bp); STRA6 (forward, 5′- TGTTGGATGAGCTTCAGTGC-3′, reverse, 5′- TGGTTCCCAGGAAGAAGATG-3′) (85 bp); HSD17B2 (forward, 5′- TGGAACTGTGGAGGTCACAA-3′, reverse, 5′- CCACTTGGAAAGCTCCAGTC-3′) (178 bp); TBP (forward, 5′-TGCACAGGAGCCAAGAGTGAA-3′, reverse, 5′-CACATCACAGCTCCCCACCA-3′) (132 bp). The PCR primers for NR5A1 and TBP were synthesized according to a previous report [16], [17]. The primers for STAR, STRA6 and HSD17B2 were designed using the Primer3 software program (frodo.wi.mit.edu). Shuttle PCR was performed in 40 cycles as follows: pre-incubation for 10 sec at 95°C, denaturation for 5 sec at 95°C and annealing/extension for 20 sec at 60°C. All samples were run in duplicate. The melting curves of the products were obtained after cycling by a stepwise increase in temperature from 55–95°C. At the end of 40 cycles, the reaction products were separated electrophoretically on an agarose gel and stained with ethidium bromide for visual confirmation of PCR products.

### Sodium bisulfite sequencing

The detailed DNA methylation status in NR5A1, STAR, STRA6 and HSD17B2 genes was investigated using the sodium bisulfite sequencing method in a pair of euESCa and choESC from one individual, the samples of which had been also analyzed by the Infinium method. The sodium bisulfite treatment of genomic DNA and the subsequent purification were carried out using EpiTect (QIAGEN) according to the manufacturer's protocol. The bisulfite modified DNA template was prepared in a 50 µl PCR mix using EpiTaq HS (TAKARA). The following primers were used: NR5A1 (forward, 5′- GTAAATGAAGAGAAATATTAATAAAGAAGG-3′, reverse, 5′-AAAAATAACAATAAACACCAAAAATCC-3′) (252 bp); STAR (forward, 5′- TAGATAAAGTTATTGGYGGGAAAGT-3′, reverse, 5′- AAAACAACAACCCAAACCTCTAAC-3′) (360 bp); STRA6 region A (forward, 5′- TGGGGGAGGAGGTTTTAGTATTAT-3′, reverse, 5′- CACATATTACCTCCTTCATAAAAACC-3′) (287 bp); STRA6 region B (forward, 5′- GGAGGAAGGAGTTGTAGAGATGAA-3′, reverse, 5′- ACTCCTAACCACACCCTTACAAAA-3′) (239 bp); HSD17B2 (forward, 5′- AAAATAGAAGGTGTGTGTTTGTGG-3′, reverse, 5′- ACACCTTCAAATACTCAAAACCAA-3′) (285 bp). The primer sequences were designed using the Primer3 software program. The primer sets covered the CpG sites analyzed by the Infinium method. All PCR studies were performed for 40 cycles as follows: denaturation for 30 sec at 94°C, annealing for 30 sec at 58°C and extension for 30 sec at 72°C. The PCR products were separated on agarose gels, excised, purified and cloned into the pGEM-T easy vector (Promega, Madison, WI). Recombinant plasmid DNA from the individual bacterial colonies were purified and sequenced to determine the presence of methylated cytosines.

### Methylation-sensitive high resolution melting analysis (MS-HRMA)

To validate the results of the Infinium and sodium bisulfite sequencing methods, the DNA methylation status of the NR5A1, STAR, STRA6 and HSD17B2 genes was examined by MS-HRMA [18], [19] using a larger number of samples of isolated euESCa (n = 7) and choESC (n = 6). MS-HRMA was carried out on a Roter-Gene 6000 thermocycler (Corbett Research, Mortlake, Australia). Briefly, 10 ng of bisulfite-modified DNA template was prepared in a 25 µl PCR mix using an EpiTect HRM PCR kit (QIAGEN). The primer sets were the same as those used for bisulfite sequencing and MS-HRMA except for those for HSD17B2. For HSD17B2, the following primers were used: HSD17B2 (forward, 5′- AGGTGTGTGTTTGTGGGTGAGTA-3′, reverse, 5′- TTCAACATAAATACCAAAACACTTCC-3′) (101 bp). PCR was performed in 40 cycles as follows: pre-incubation for 5 min at 95°C, denaturation for 10 sec at 95°C, annealing for 30 sec at 55°C and extension for 10–26 sec (primer-dependent) at 72°C. The methylation-sensitive high resolution melting was run in the interval of 72°C to 95°C with 2 sec, 0.1°C steps, acquiring fluorescence data at the Roter-Gene HRM channel. All reactions were run in duplicate.

### Statistical analysis

Real-time PCR data were subjected to a Mann-Whitney test. A value of *p*<0.05 was considered to be statistically significant. The data are presented as the means ± SD.

## Results

### Genome-wide DNA methylation profiling

As shown in Figure S1, the histograms of each group show very similar distribution patterns.

**Figure 1:**
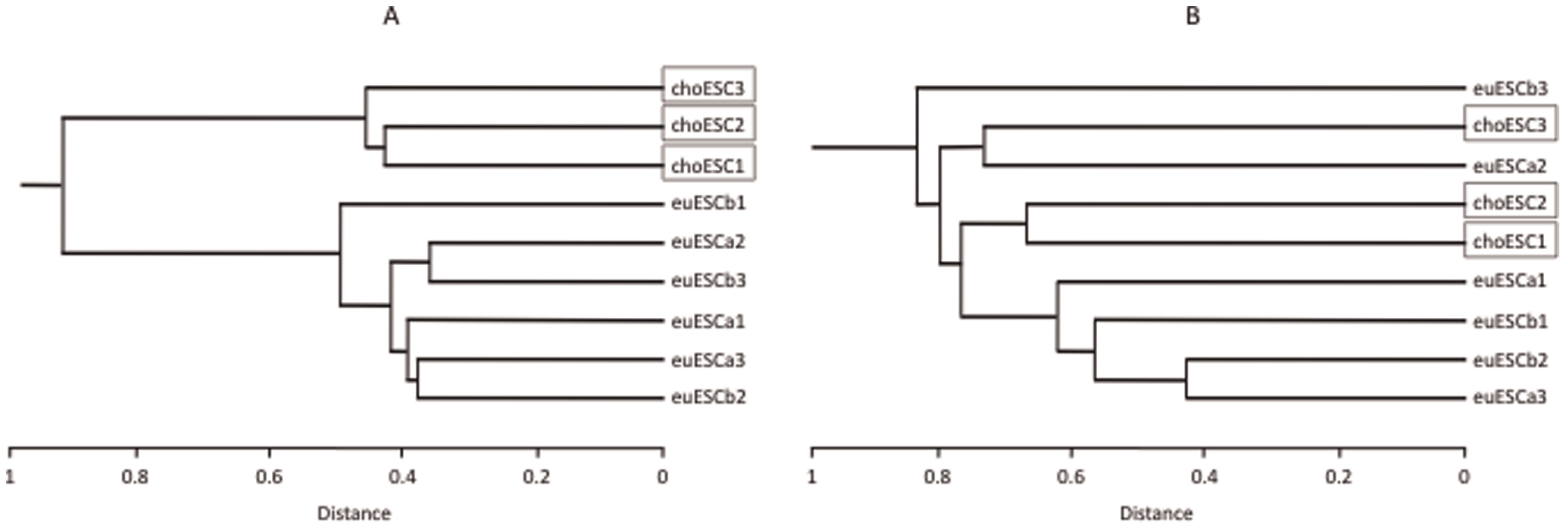
The results of the hierarchical clustering analysis of genome-wide DNA methylation status (A) and the transcriptome analysis (B). A hierarchical dendrogram showing that the choESC samples cluster according to lineage and were separated from the euESC samples in DNA methylation (A), despite the fact that no cluster formations can be recognized in the transcriptome analysis (B).

**Figure 2:**
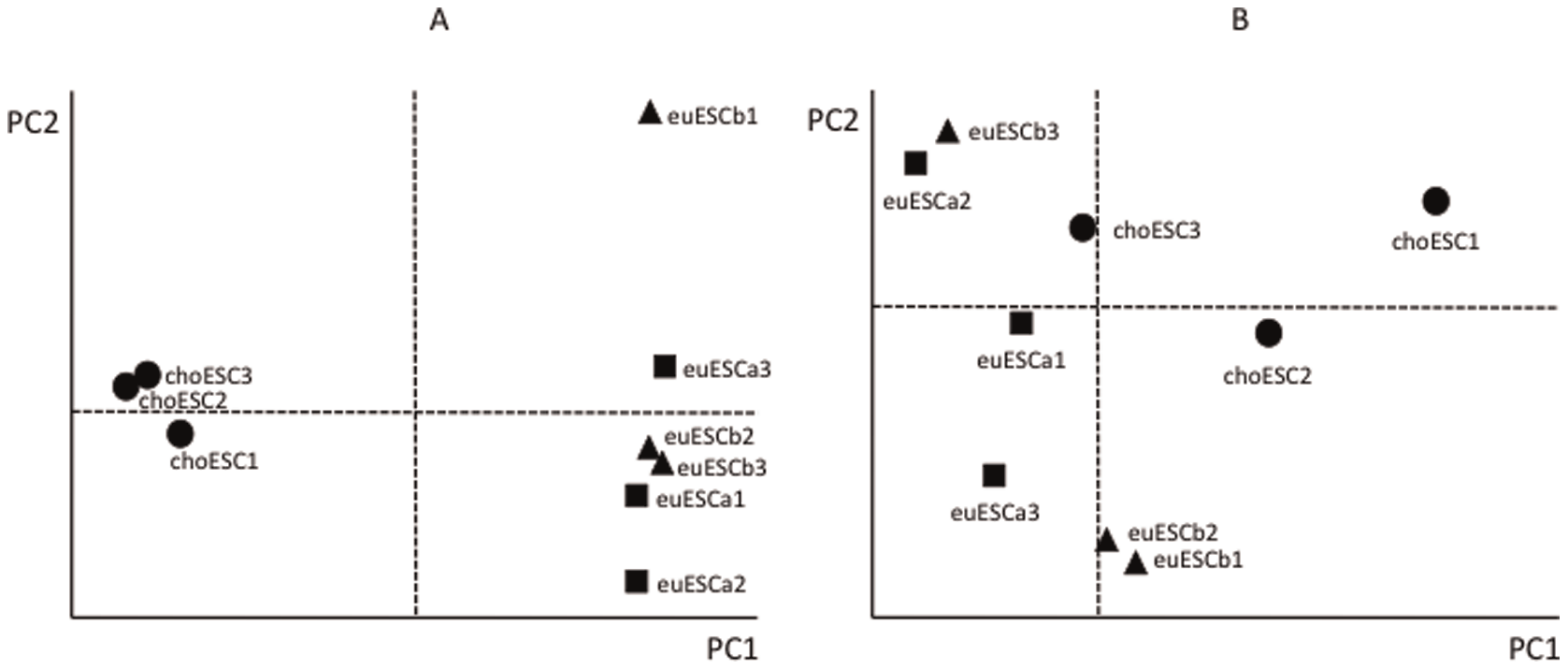
The results of the principal component analysis of genome-wide DNA methylation status (A) and the transcriptome analysis (B). The closed squares, closed triangles and closed circles represent euESCa, euESCb and choESC, respectively. The PC1 axis clearly distinguished the choESC from the euESC, whereas the euESCa and euESCb were identical in DNA methylation (A). No clear clusters were observed in the transcriptome analysis (B).

To identify the CpGs that showed significant methylation changes in the euESCb or choESC relative to the euESCa, the average β value of each CpG and the differences between each group were calculated. CpGs expressing over 0.2 were then abstracted. 515 CpGs (441 genes) were hypomethylated and 368 CpGs (329 genes) were hypermethylated in the choESC, while only nine CpGs were hypomethylated and six CpGs were hypermethylated in the euESCb (data not shown). The hierarchical dendrogram was divided in two clusters: choESC and euESC. The euESCa and euESCb were not further subdivided (Figure 1A). These findings were also supported by PCA. PCA clearly separated the choESC cells from eutopic ESC, while no clear separation was observed between the euESCa and euESCb (Figure 2A).

To determine the biological relevance of the differentially methylated genes, GO and KEGG pathways analyses were performed. The differently hypomethylated genes in the choESC, compared to that observed in the euESCa, were related to the biological processes of signal transduction and development and the molecular functions of signaling molecules and cell adhesion molecules. Other significant related terms were detected as illustrated. In the KEGG pathway analysis, genes related to cytokine-cytokine receptor interaction were hypomethylated (Table 1). On the other hand, genes differentially hypermethylated in the choESC, compared to that observed in the euESCa, were related to the biological processes of signal transduction and development and the molecular functions of receptors and signaling molecules. In the KEGG pathway analysis, genes related to cytokine-cytokine receptor interaction were also hypermethylated (Table 2).

**Table 1:** Hypomethylation in choESC compred to euESCa.

*Biological Process*		
Term	count	p-value
Signal transduction	96	0.003088
Developmental processes	72	0.000090
Cell surface receptor mediated signal transduction	54	0.002227
Immunity and defense	46	0.001015
Cell communication	40	0.006518
Transport	38	0.045504
Ectoderm development	34	0.000014
Neurogenesis	32	0.000005
Lipid, fatty acid and steroid metabolism	29	0.003795
Mesoderm development	22	0.010419
Cell adhesion	21	0.038915
Carbohydrate metabolism	20	0.043741
Amino acid metabolism	12	0.011551
Receptor protein tyrosine kinase signaling pathway	10	0.036862
Macrophage-mediated immunity	9	0.011863
Segment specification	8	0.048202
Cytokine/chemokine mediated immunity	7	0.048202
Other lipid, fatty acid and steroid metabolism	5	0.002386

*Molecular Function*		
Term	count	p-value
Signaling molecule	35	0.000106
Cell adhesion molecule	15	0.044374
Growth factor	9	0.005815
Other cell adhesion molecule	7	0.033738

*KEGG pathway*		
Term	count	p-value
Cytokine-cytokine receptor interaction	14	0.014843
Melanoma	6	0.033369
Tyrosine metabolism	5	0.024531
Type II diabetes mellitus	5	0.030423
Phenylalanine metabolism	4	0.017268

**Table 2:** Hypermethylation in choESC compred to euESCa.

*Biological Process*		
Term	count	p-value
Signal transduction	78	0.000140
Developmental processes	47	0.010955
Cell surface receptor mediated signal transduction	39	0.008625
Immunity and defense	34	0.003117
Cell communication	30	0.010674
Other receptor mediated signaling pathway	9	0.019354
Cytokine and chemokine mediated signaling pathway	9	0.039747
Blood circulation and gas exchange	6	0.010895

*Molecular Function*		
Term	count	p-value
Receptor	38	0.008257
Signaling molecule	22	0.017587
Cytoskeletal protein	21	0.046184
Extracellular matrix	15	0.003210
Defense/immunity protein	13	0.013327
Serine protease inhibitor	8	0.000282
Immunoglobulin receptor family member	8	0.002151
Protease inhibitor	8	0.005002
Cytokine receptor	5	0.047735
Non-receptor tyrosine protein kinase	4	0.027526
Other ion channel	4	0.033505

*KEGG pathway*		
Term	count	p-value
Cytokine-cytokine receptor interaction	12	0.004707
Apoptosis	7	0.03516
Insulin signaling pathway	7	0.027340
Hematopoietic cell lineage	6	0.015370

Lists of statistically significant GO terms (Biological process and molecular function) and KEGG pathway terms in hypomethylated genes (Table 1) and in hypermethylated genes (Table 2) in choESC compared to euESCa.

### Expression of epigenetic chromatin modification enzymes

The results of the real-time PCR array are shown in Table S1 and Figure S2. If genes inducing more than a 2-fold change and *p* values of less than 0.01 are significant, then none of the tested genes, including DNA methyltransferases, showed significant differences between the euESCb and euESCa or between the choESC and euESCa.

### Transcriptome analysis

Of the 28,869 human genes identified in our gene index, 498 were upregulated and 329 were downregulated in the choESC compared to that observed in the euESCa. Two genes were upregulated and eight genes were downregulated in the euESCb compared to that observed in the euESCa (data not shown).

GO and KEGG pathways analyses were performed as DNA methylation analyses of the selected genes (Table S2 and S3). There are numerous listed terms in both up- and downregulated genes; however, only several terms were concordant with the results of the genome-wide DNA methylation analysis.

### Combination of transcriptome and methylation analyses

Hierarchical clustering and PCA were performed on the euESCa and choESC. In the DNA methylation analysis, hierarchical clustering separated the samples of the choESC from the others using filtered genes in the DNA methylation analysis (Figure 1A). PCA also segregated the samples from the choESC (Figure 2A). Interestingly, neither hierarchical clustering nor PCA showed clear cluster formations using differently expressed genes in the transcriptome analysis (Figure 1B and Figure 2B). In PCA, even after the axes were increased up to 10 with their combination, there was no cluster formation (data not shown). PCA or cluster analysis is the task of grouping a set of objects so that more similar objects are segregated from the other objects by mathematical procedures. Thus, these analyses can visualize the extraneous group in mixed populations, but are not usually used to identify the latent difference. Therefore, the absence of cluster formation of choESC does not mean that there is no difference in gene expression patterns between choESC and euESC. In fact, as shown in Table S2 and S3, there are a number of differentially expressed genes in choESC compared with euESCa.

The comparative analysis of the euESCa and choESC is shown in Table S4, S5, S6 and S7. Correlations between DNA methylation and mRNA expression were observed in 75 filtered genes (22 genes: hypermethylation – low expression, 32 genes: hypomethylation – high expression, 10 genes: hypomethylation – low expression, 11 genes: hypermethylation – high expression). In some of the combinations, significant GO and KEGG terms were listed.

### mRNA expression levels of the NR5A1, STAR, STRA6 and HSD17B2 genes

**Figure 3:**
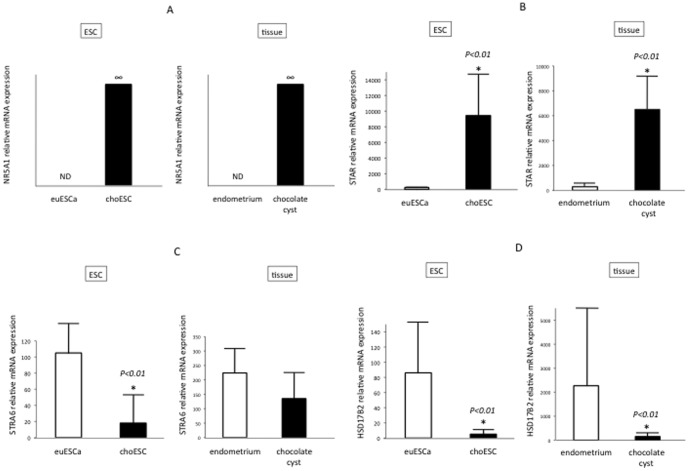
The mRNA levels of NR5A1 (A), STAR (B), STRA6 (C) and HSD17B2 (D) in ESCa, choESC, eutopic endometrium and chocolate cysts. ESCa (n = 7), choESC (n = 5), eutopic endometria (n = 17) and chocolate cysts (n = 6) were subjected to total RNA isolation followed by real-time RT-PCR. The relative mRNA expression normalized to that of TBP (an internal control) was calculated. The values are the means ±SD. *, *p*<0.01. ND: not detected.

Of the differentially-methylated and expressed genes in choESC compared with euESCa, we focused on the steroidogenesis-related genes, NR5A1, STAR, STRA6 and HSD17B2. To validate the results of the transcriptome analysis, real-time RT-PCR was conducted on these genes using a larger number of samples, including isolated euESCa (n = 7) and choESC (n = 5), endometrial tissues (n = 17) and chocolate cysts (n = 6). The NR5A1 mRNA levels were remarkably high in both the choESC and chocolate cysts, whereas NR5A1 mRNA expression was not detected in the euESCa and eutopic endometrium (Figure 3A). The STAR mRNA levels were significantly higher in both the choESC and chocolate cysts than those in the euESCa and eutopic endometrium (Figure 3B). The STRA6 mRNA levels were significantly lower in the choESC than those in the euESCa, and there were no significant differences between the levels in the eutopic endometrium and chocolate cysts (Figure 3C). The HSD17B2 mRNA levels were significantly lower in both the choESC and chocolate cysts than those in the euESCa and eutopic endometrium (Figure 3D). These results were consistent with the data obtained from the transcriptome analysis.

### DNA methylation analysis of the NR5A1, STAR, STRA6 and HSD17B2 genes

**Figure 4:**
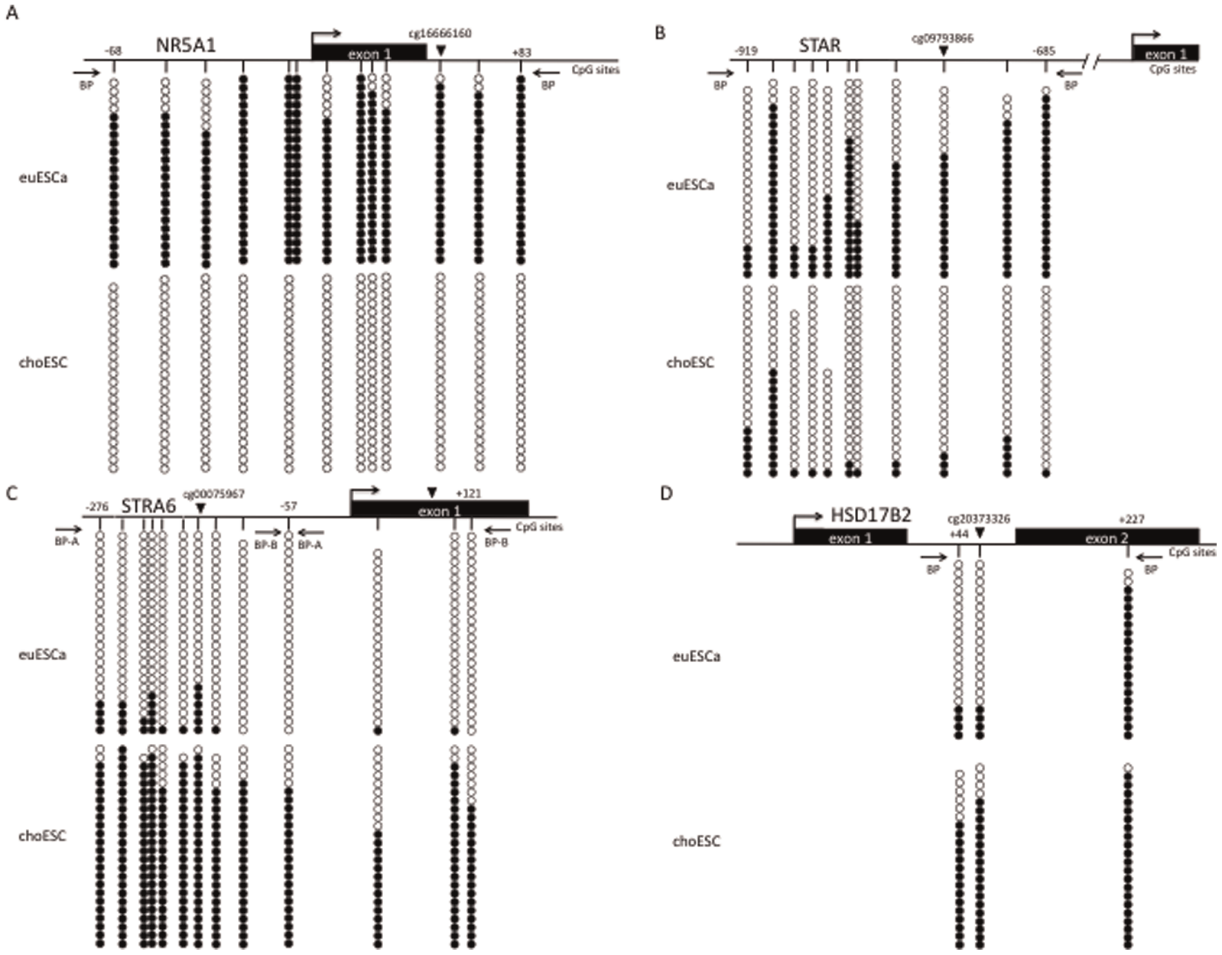
The results of the sodium bisulfite sequencing analyses of NR5A1 (A), STAR (B), STRA6 (C) and HSD17B2 (D) in the euESCa and choESC samples. The DNA methylation profile in the genomic regions of the NR5A1, STAR, STRA6 and HSD17B2 genes was analyzed by a sodium bisulfite sequencing method in a pair of euESCa and choESC from one individual, which had already been analyzed by the Infinium method. In NR5A1 and STRA6, the DNA methylation status of the proximal promoter and first exon was analyzed. The primer pairs BP-A and BP-B amplify region A and B, respectively, in STRA6. In STAR, the distal promoter region was analyzed. In HSD17B2, the first intron and second exon region were analyzed. The arrows indicate the positions of the bisulfite primers. Closed triangles represent the CpG sites analyzed by the Infinium method, and are accompanied by the identification names. •, methylated CpG sites; ◯, unmethylated CpG sites; BP, bisulfite primer.

The DNA methylation status in the genomic regions of the NR5A1, STAR, STRA6 and HSD17B2 genes was investigated by the sodium bisulfite sequencing method (Figure 4). In NR5A1 and STAR, the CpG sites were hypomethylated in choESC compared to euESCa (Figure 4A and 4B), whereas the CpG sites in STRA6 and HSD17B2 were hypermethylated (Figure 4C and 4D). These results were consistent with the data from the Infinium analysis.

**Figure 5:**
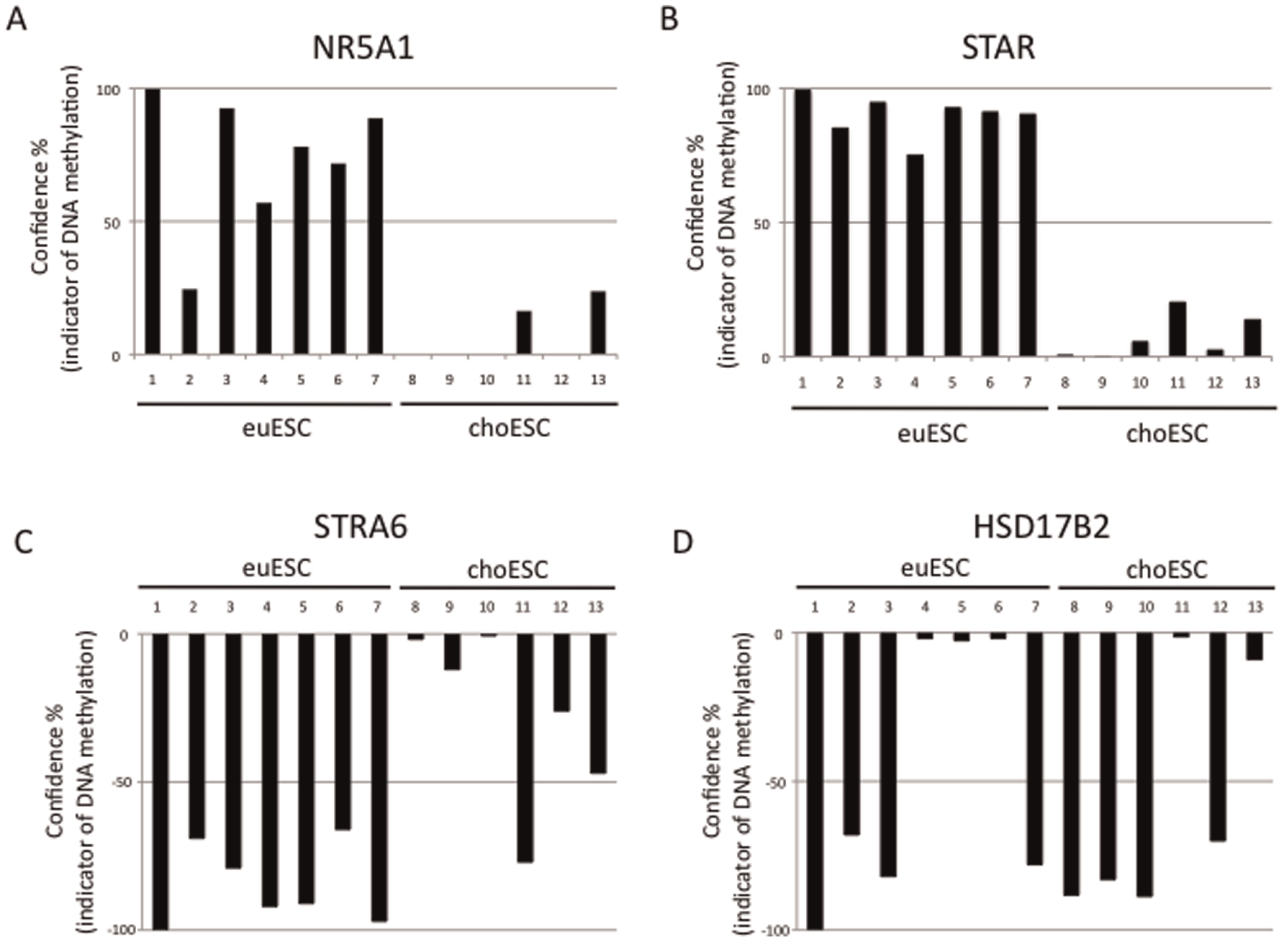
The DNA methylation status as determined by the methylation-sensitive high resolution analyses (MS-HRMA) of NR5A1 (A), STAR (B), STRA6 (C) and HSD17B2 (D) in euESCa and choESC. Sample No.-wide DNA methylation and transpciptome as euESCa, and sample No. 8, 9, 10 were also analyzed as choESC. Sample No. 1 and 10 were analyzed for bisulfite sequencing as euESCa and choESC, respectively. The confidence value calculated by MS-HRMA indirectly indicates the DNA methylation level. The DNA methylation status of euESCa-1 was shown as 100% identical with regard to the DNA methylation. The confidence values of each sample were calculated in comparison with euESCa-1. In NR5A1 and STAR, the confidence values were lower in choESC than those in euESCa, indicating that the choESC are hypomethylated compared with the euESCa. In STRA6 and HSD17B2, the DNA methylation status of euESCa-1 was shown as −100% (arbitrary defined reverse axis value) identical regarding DNA methylation, indicating a DNA hypomethylation status. In STRA6, the confidence values were higher in choESC than those in euESCa, indicating that the choESC are hypermethylated compared with the euESCa. In HSD17B2, the DNA methylation status varied among individuals with euESCa and choESC. The samples of euESCa and choESC were isolated from seven and six patients, respectively.

We further analyzed the DNA methylation status on these genes by MS-HRMA (Figure 5). NR5A1 and STAR showed hypomethylation in choESC compared with euESCa (Figure 5A and 5B), which was consistent with the results of the Infinium results. However, the STRA6 gene showed hypermethylation in choESC compared with euESCa, with a few exceptions (Figure 5C), which was also consistent with Infinium results. In HSD17B2, the DNA methylation status varied among individuals in both euESCa and choESC (Figure 5D). We speculate that there may be several explanations for the discrepancy between the Infinium and MS-HRMA results. First, the HSD17B2 primer set used for MS-HRMA only covers two CpGs, which may result in difficulties detecting significant differences between two samples. Second, the PCR-amplified region was located in the first intron. This region might not be important with regard to the gene expression, and there is a possibility that the DNA methylation status in this region may be mutable, depending on each individual. Therefore, these results are reasonable.

## Discussion

Recently, Borghese B. *et al.* reported genome-wide DNA methylation profiling in several subtypes of endometriotic tissues, using MeDIP-chip technology to focus on promoters [20]. They showed that numerous methylation differences exist compared to eutopic endometria, and their reports provide meaningful new information regarding epigenetic status using precious clinical materials. Meanwhile, our experimental strategy is aimed at revealing more precise genome-wide DNA methylation profiles using the Infinium Beadchip in cultured homogeneous endometrial stromal cells derived from both eutopic endometria with or without endometriosis and ovarian endometrial cysts.

In comparison with eutopic ESC, numerous CpGs in choESC obtained from endometrial cysts are methylated differently, both high and low. Based on the data obtained from the methylation profiles, we determined the specific functions in the choESC using a robust in silico analysis. According to the GO and pathway analyses using the filtered genes, interesting terms occupy the top of these lists in terms of the pathogenesis and development of endometriosis. Namely, there is aberrant epigenetic status in the genes associated with receptors, signaling pathways and immune responses, which suggests that choESC express aberrant behavior in peritoneal circumstances. It is also interesting that choESC indicated abnormal differentiation, such as neurogenesis, with the lowest *p* value. This means choESC has already differentiated into other cell types, and choESC have intriguing footprints that imply the presence of abnormal developmental processes. Despite the fact that not all of the terms were identical in the DNA methylation and transcriptome analyses, many of the terms were commonly different. Therefore, the results of these analyses raise the possibility that epigenetic modification of DNA may be involved in the pathogenesis of endometriosis.

We also investigated the differences regarding genome-wide DNA methylation and transcriptome analyses between eutopic ESCs with and those without endometriosis. Researchers have described that eutopic endometria from endometriotic females have different characteristics than eutopic endometria from non-affected females in terms of inflammation, steroidogenesis [21], migratory behavior [22] and sensitivity to apoptosis [23]. On the contrary, our study did not find any meaningful differences. A small number of candidate genes expressed different values (data not shown); however, known important genes associated with steroidogenesis, inflammation and apoptosis were not included. One of the novelties of this study is that primary cultured ESC was used for analysis. After ESC was isolated and cultured for several days, DNA methylation status and mRNA expression levels were analyzed. We speculate that influences by the pelvic environment may have been removed by in vitro culture. In other words, aberrant gene expression in eutopic ESC with endometriosis may be rather due to the influence by pelvic inflammatory environment than due to aberrant DNA methylation or gene mutations. This may be also supported by our result that there was no clear difference in DNA methylation status between eutopic ESC with and without endometriosis.

Of the various differentially methylated and/or expressed genes in choESC compared with eutopic ESC, we focused on the steroidogenesis-related genes, NR5A1, STAR, STRA6 and HSD17B2. A further analysis of the mRNA expression by real-time RT-PCR in these genes validated the accuracy of the results of the transcriptome analysis using a larger number of samples. Interestingly, the aberrant expression of these steroidogenesis-related genes in choESC led us to speculate a possible mechanism of pathogenesis for endometriosis, wherein estradiol synthesis is locally enhanced within the endometriotic tissue. Remarkably high mRNA expression and DNA hypomethylation in the NR5A1 gene, encoding SF-1, was found in the choESC and chocolate cysts in this study, which is consistent with the previous reports [2], [24]. Since SF-1 is a transcription factor that induces the expression of STAR and cyp19a1, which encode aromatase, the high SF-1 expression may have contributed to the high mRNA expression of STAR and cyp19a1 in the choESC and chocolate cysts. Higher cyp19a1 mRNA expression in choESC was found in the transcriptome analysis of this study (data not shown). In addition, our results showed that there was low expression of the STRA6 and HSD17B2 genes in the choESC and chocolate cysts. Pavone et al. also reported that low expression of STRA6 in endometriotic stromal cells is associated with a reduced amount of active retinoic acids, which in turn, decreases the HSD17B2 expression in endometriotic epithelial cells [25]. STRA6 is an essential cell surface receptor for retinol binding protein, and is necessary for the retinol uptake into cells. HSD17B2 converts estradiol into estrone. Thus, low expression of STRA6 and HSD17B2 results in the enhanced endogeneous synthesis of estradiol. Regarding the mechanism responsible for the locally enhanced estradiol synthesis in the endometriotic lesion, besides the report by Blun et al. showing that increased SF-1 expression was induced by aberrant DNA hypomethylation in the SF-1 promoter region, which causes aberrant aromatase expression, the present study proposed additional mechanisms; aberrant DNA hypomethylation in the NR5A1 gene encoding SF-1, and aberration of retinoic acid metabolism, which is due to the low STRA6 expression induced by aberrant DNA hypermethylation. Taken together, the aberrant expression of these steroidogenesis-related genes causes enhanced estradiol synthesis within the endometriotic tissue, which is involved in the development of endometriosis. A further analysis of the DNA methylation by independent methods on these genes validated the accuracy of the array-based results of the Infinium method using a larger number of samples. NR5A1 and STAR showed DNA hypomethylation, and STRA6 and HSD17B2 showed hypermethylation in the choESC samples compared with the euESCa samples. These findings were consistent with the Infinium results, suggesting that aberrant DNA methylation in the key steroidogenesis-related genes causes aberrant gene expression, leading to the development of endometriosis.

The characterization of eutopic and ectopic ESC using clustering analysis and PCA provides interesting information. Both analyses showed that choESC were clearly segregated from eutopic ESC in the DNA methylation analysis, although no clear segregation was observed in the transcriptome analysis. The same analyses were also performed using transcriptome data previously reported by Borghese B. and Eyster KM. [6], [26]. Interestingly we could not find any separation of ectopic endometrial from eutopic ones in both data (data not shown), which is in agreement with our result. These findings indicate the significance of DNA methylation analysis for the identification of endometriotic cells. To elucidate the mechanisms underlying the development of endometriosis, genome-wide DNA methylation analysis might be a superior approach to transcriptome analysis. Although transplantation theory is most acceptable so far in pathogenesis of endometriosis, we speculate coelomic metaplasia theory is not preclusive according to out observations. It may not be plausible for endometrial differentiated cells to initiate self-regenerating accompanied by the DNA methylation alteration.

Epigenetic marks are rewritable by a series of enzymes that modify histones and DNA. With regard to DNA methylation, transmethylation is directly performed by DNA methyltransferases (Dnmts). Wu *et al.* described the presence of elevated Dnmt1, Dnmt3a and Dnmt3b in the epithelial components of endometriotic implants compared to that observed in eutopic endometria without endometriosis [27]. On the other hand, only Dnmt3a was found to be upregulated in eutopic endometria in females with endometriosis compared to that observed in endometriosis-free females. The results of their investigation suggest that hypermethylation may frequently occur in the epithelium in endometriosis. Of interest, they also depicted the protein expression contrasts between epithelial and stromal cells in eutopic and ectopic endometria using immunostaining, and the Dnmts expression levels were found to be much lower in the ESC than the endometrial epithelial cells. In our experiment, the quantitative PCR array-based method showed no statistically significant differences in Dnmts mRNA expression between eutopic ESC with and those without endometriosis or between eutopic and ectopic ESC. Since, as is widely recognized, the recruitment of Dnmts onto target genomic sites is more important than its expression level, abnormal DNA methylation should occur in situations without aberrant Dnmts expression. Meanwhile, histone modification enzyme expression, which provides other critical epigenetic memories, also showed no differences between groups.

Observations of the relationship between the DNA methylation profiles and transcriptome analyses disclosed not only the expected positive correlations (hypermethylation/low expression and hypomethylation/high expression), but also some negative correlations (hypermethylation/high expression and hypomethylation/low expression). To describe the intimate association between DNA methylation and mRNA expression is impossible because the specification of the Beadchip covers only approximately half of the genes and analyzes only two CpGs in one gene. There may be complicated interactions and networks between DNA methylation and transcription in genome-wide level observations.

In summary, this study provided fundamental DNA methylation data concerning endometriosis using non-treated cultured ESC. It is appealing to establish wider profiles of whole epigenomes, including both DNA methylation and histone modifications. We believe that this type of novel information would be useful for future research regarding therapeutic strategies and preventative medicine for treating endometriosis.

## Supporting Information

Figure S1 **Histograms of the β values for the euESCa (open column), euESCb (shaded column) and choESC (closed column).**
(TIF)

Figure S2 **A volcano plot of the** ***p*** **values correlated with the fold changes in gene expression in the euESCb (A) and choESC (B) relative to the euESCa, as measured using qRT-PCR.** The open circles represent DNA methyltransferases.
(TIF)

Table S1 **Of the 84 genes, genes expressing greater than 2-fold or less than 0.5 were not recognized except KATB2 in transcriptome analysis using GeneChip.** GeneChip analysis revealed KAT2B expression in choESC was 2.55-fold higher (p<0.0034) compared to euESCa.
(DOCX)

Table S2 **Lists of statistically significant GO terms (biological process and molecular function) and KEGG pathway terms in high expressed genes in choESC compared to euESCa.**
(DOCX)

Table S3 **Lists of statistically significant GO terms (biological process and molecular function) and KEGG pathway terms in low expressed genes in choESC compared to euESCa.**
(DOCX)

Table S4 **Lists of genes that have significant hypermethylated CpGs with significant low mRNA expressions in choESC compared to euESCa.** The significant GO terms were shown below the gene lists.
(DOCX)

Table S5 **Lists of genes that have significant hypomethylated CpGs with significant high mRNA expressions in choESC compared to euESCa.** The significant GO and KEGG pathway terms were shown below the gene lists.
(DOCX)

Table S6 **Lists of genes that have significant hypo-methylated CpGs with significant low mRNA expressions in choESC compared to euESCa.** The significant GO terms were shown below the gene lists.
(DOCX)

Table S7 **Lists of genes that have significant hypermethylated CpGs with significant high mRNA expressions in choESC compared to euESCa.**
(DOCX)
