# Supplementary material for: Genome-Wide DNA Methylation Profiling in Cultured Eutopic and Ectopic Endometrial Stromal Cells
Source: PLoS One. 2014 Jan 23;9(1):e83612. doi: 10.1371/journal.pone.0083612 (PMC3900404; doi:10.1371/journal.pone.0083612)
Supplement: Table S1 — Of the 84 genes, genes expressing greater than 2-fold or less than 0.5 were not recognized except KATB2 in transcriptome analysis using GeneChip. GeneChip analysis revealed KAT2B expression in choESC was 2.55-fold higher (p<0.0034) compared to euESCa. (DOCX) [file pone.0083612.s003.docx]

Table S1. Results of real-time PCR array. mRNA expression of epigenetic modification enzymes in euESCb and choESC compared to euESCa.

|  | euESCb | | choESC | |
| --- | --- | --- | --- | --- |
| symbol | Fold change | p-value | Fold change | p-value |
| ASH1L　　　　　ATF2　　　AURKA　　AURKB　　AURKC　　CARM1　　CDYL　　　　　CIITA　　CSRP2BP　　DNMT1　DNMT3A　　DNMT3B　　DOT1L　　　DZIP3　　EHMT2　　ESCO1　　ESCO2　　　　HAT1　　　HDAC1　　HDAC10　　HDAC11　　HDAC2　　HDAC3　　HDAC4　　HDAC5　　HDAC6　　HDAC7　　HDAC8　　HDAC9　　KDM1　　KDM5B　　KDM5C　　KDM4A　　KDM4C　　KDM6B　　KAT2A　　KAT2B*　　KAT5　　　　　MBD2　　　　　　　MLL　　　　　　　MLL3　　　　　MLL5　　　MYSM1　　MYST1　　　MYST2　　　MYST3　　　MYST4　　　NCOA1　NCOA3　NCOA6　　　NEK6　　　　　　NSD1　　　　　　　　PAK1　　　　　PRMT1　　　PRMT2　　PRMT3　　PRMT5　　　PRMT6　　　PRMT7　　　PRMT8　　　　RNF2　　　　　RNF20 | 1.0923　　　　　1.1761　　　　　　1.3355　　　　　　2.1595　　　　　-1.5977　　　　1.1788　　　　　1.1761　　　　　-1.1508　　　　-1.0253　　　　　　1.031　　　　　　　1.1653　　　　　　1.5772　　　　　　　1.226　　　　　　　-1.0541　　　　　1.0381　　　　　　　　　1.29　　　　　　　　　　2.3685　　　　　　　　1.284　　　　　　　1.1439　　　　　　-1.0158　　　　-1.0253　　　　　　1.389　　　　　　　1.1519　　　　　-1.0372　　　　　1.2547　　　　　　　1.123　　　　　　　1.1282　　　　　　1.0673　　　　　　1.0405　　　　　　1.4413　　　　　　　1.226　　　　　　　1.4513　　　　　　1.0168　　　　　　1.1101　　　　　　1.1075　　　　　　1.0454　　　　　　-1.4399　　　　　　1.136　　　　　　　1.1815　　　　　　1.1413　　　　　-1.0372　　　　　1.3698　　　　　1.1734　　　　　-1.0396　　　　　1.0624　　　　　1.2518　　　　　1.5411　　　　　1.1734　　　　　-1.0348　　　　　1.1815　　　　　-1.0787　　　　-1.0088　　　　　1.2663　　　　　1.1599　　　　　-1.0112　　　　　1.0673　　　　　1.1439　　　　　　　1.293　　　　　　　1.0381　　　　　1.1204　　　　　1.0454　　　　　　　1.187 | 0.831856　　0.704106　　0.458967　0.076896　0.588402　0.622934　0.770815　0.885992　0.731932　0.948226　0.769811　0.454567　0.690519　0.784103　0.987396　0.67984　0.173932　0.166984　0.878391　0.887839　0.907559　0.723522　0.690259　0.758511　0.974327　0.983206　0.815679　0.790896　0.996775　0.175912　0.680244　0.66322　0.999885　0.893156　0.954265　0.977228　0.110626　0.878863　0.400823　0.819892　0.820212　0.621902　0.807434　0.837075　0.903574　0.70184　0.633988　0.975456　0.820824　0.723214　0.599699　0.838529　0.859731　0.47566　0.907807　0.913494　0.709699　0.315344　0.891658　0.824431　0.846362　0.796428 | -1.3454　　　-1.2728　　　-1.1339　　　　1.0634　　　　-1.1366　　　-1.6298　　　　-1.549　　　　　　-2.247　　　　　-1.1931　　-1.2466　　　-1.6795　　　-1.0752　　　-1.7227　　　-1.0149　　　-1.1471　　　-1.2153　　　　1.3648　　　　-1.1658　　　-1.3547　　　-1.1418　　　-1.5312　　　-1.7387　　　-1.2553　　　-1.5526　　　-1.8167　　　-1.6563　　　-1.6525　　　-1.3423　　　-1.5171　　　-1.5999　　　-1.7467　　　-2.2212　　　-1.4654　　　-1.2582　　　　　　-2.76　　　　　　-1.2699　　　　1.3275　　　　　　-1.396　　　　-1.1287　　　-1.5889　　　-1.6298　　　-1.7187　　　-1.5032　　　-1.5206　　　-1.2069　　　　-1.699　　　　-3.9032　　　-1.0802　　　-1.5383　　　-1.4893　　　　1.0832　　　　-1.4722　　　　-1.5419　　　-1.1183　　　-1.1392　　　　1.0154　　　　-1.0928　　　-1.2323　　　-1.4057　　　　-2.425　　　　　-1.1903　　　-1.3025 | 0.308392　0.381178　0.693793　0.825615　0.616548　0.138827　0.237367　0.254627　0.535244　0.417197　0.184364　0.737715　0.188082　0.649505　0.534858　0.499981　0.676172　0.380354　0.383138　0.595377　0.172565　0.296648　0.355061　0.257461　0.34772　0.362947　0.181037　0.170848　0.419357　0.130225　0.19013　0.20769　0.209307　0.532321　0.091468　0.499303　0.23056　0.358372　0.44002　0.273639　0.249109　0.267327　0.36726　0.147216　0.430376　0.21807　0.400792　0.66042　0.16621　0.24077　0.684634　0.291414　0.392466　0.645115　0.412872　0.858453　0.631842　0.266278　0.400951　0.261876　0.282093　0.439429 |
| RPS6KA3　RPS6KA5　SETD1A　　SETD1B　　SETD2　　　SETD3　　　SETD4　　　SETD5　　SETD6　　　SETD7　　　SETD8　　SETDB1　　SETDB2　　SMYD3　　SUV39H1　　SUV420H1　　UBE2A　　UBE2B　　USP16　　　USP21　　　USP22 WHSC1 | 1.0215　　　　　1.3857　　　　　1.0847　　　　　　　1.187　　　　　　　1.1572　　　　　1.1178　　　　　1.1075  1.2693　　　　　　-1.042　　　　　　1.0673　　　　　　1.4116　　　　　　1.0551　　　　　1.3986　　　　　1.1152　　　　　　　1.305　　　　　　　1.0999　　　　　1.0649　　　　　1.1898　　　　　1.1519　　　　　1.0822　　　　　1.1439　　　　　1.4479 | 0.948605　0.885047　0.922732　0.863994　0.768857　0.445772　0.923806　0.695037　0.864191　0.830854　0.609313　0.976319　0.798596　0.38543　0.197804　0.929554　0.842965　0.392859　0.724675　0.949594　0.883327 0.250103 | -1.5312　　　-3.0133　　　　-1.452　　　　-1.6951　　　-1.3423　　　-1.2728　　　-1.1685　　　-1.7068　　　-1.9697　　　　-1.264　　　　-1.6795　　　-1.4254　　　-1.5852　　　-1.2728　　　　1.0487　　　　-1.5962　　　-1.2611　　　-1.2669　　　-1.2935　　　-1.4928　　　-1.6756　　　-1.3116 | 0.108582　0.272062　0.290437　0.254473　0.344878　0.142462　0.590966　0.23763　0.234345　0.392529　0.291284　0.391213　0.389253　0.200086　0.793165　0.221901　0.261229　0.200581　0.345391　0.319567　0.212669　0.42314 |
