# Supplementary material for: Genome-Wide DNA Methylation Profiling in Cultured Eutopic and Ectopic Endometrial Stromal Cells
Source: PLoS One. 2014 Jan 23;9(1):e83612. doi: 10.1371/journal.pone.0083612 (PMC3900404; doi:10.1371/journal.pone.0083612)
Supplement: Table S2 — Lists of statistically significant GO terms (biological process and molecular function) and KEGG pathway terms in high expressed genes in choESC compared to euESCa. (DOCX) [file pone.0083612.s004.docx]

Table S2. High expression in choESC compred to euESCa

| *Biological Process* | | |
| --- | --- | --- |
| Term | count | p-value |
| Signal transduction　　　　　　　　　　　　　　　　　　　　　　　　　　　　　　　　　　　　　　　　　　　　　　　　　　　　　　　　　Developmental processes　　　　　　　　　　　　　　　　　　　　　　　　　　　　　　　　　　　　　　　　　　　　　　　　　　　　　　　　　　　　　　　　　Cell communication　　　　　　　　　　　　　　　　　　　　　　　　　　　　　　　　　　　　　　　　　　　　　　　　　　　　　　　　　　　　　　　　　　　　　　　　Immunity and defense　　　　　　　　　　　　　　　　　　　　　　　　　　　　　　　　　　　　　　　　　　　　　　　　　　　　　　　　　　　　　　　　　　　　　　　　　　　Lipid, fatty acid and steroid metabolism　　　　　　　　　　　　　　　　　　　　　　　　　　　　　　　　　　　　　　　　　　　　　　　　　Ectoderm development　　　　　　　　　　　　　　　　　　　　　　　　　　　　　　　　　　　　　　　　　　　　　　　　　　　　　　　　　　　　　　　　　　　　　　　　　　Cell adhesion　　　　　　　　　　　　　　　　　　　　　　　　　　　　　　　　　　　　　　　　　　　　　　　　　　　　　　　　　　　　　　　　　　　　　　　　　　　　　Neurogenesis　　　　　　　　　　　　　　　　　　　　　　　　　　　　　　　　　　　　　　　　　　　　　　　　　　　　　　　　　　　　　　　　　　　　　　　　　　　　　　　　　　　　　　　　Protein phosphorylation　　　　　　　　　　　　　　　　　　　　　　　　　　　　　　　　　　　　　　　　　　　　　　　　　　　　　　　　　　　　　　　　　　　　　　　　　Other metabolism　　　　　　　　　　　　　　　　　　　　　　　　　　　　　　　　　　　　　　　　　　　　　　　　　　　　　　　　　　　　　　　　　　　　　　　　　　　　　　　　　　　　　　　　　　　　　　　　　　　　　　　　　　　　　　　　　　　　　　　　　　　　　　Ligand-mediated signaling　　　　　　　　　　　　　　　　　　　　　　　　　　　　　　　　　　　　　　　　　　　　　　　　　　　　　　　　　　　　　　　　　　　　　　Cell adhesion-mediated signaling　　　　　　　　　　　　　　　　　　　　　　　　　　　　　　　　　　　　　　　　　　　　　　　　　　　　　　　　　　　Cell motility　　　　　　　　　　　　　　　　　　　　　　　　　　　　　　　　　　　　　　　　　　　　　　　　　　　　　　　　　　　　　　　　　　　　　　　　　　　　　　　　　　　　　　Cytokine and chemokine mediated signaling pathway　　　　　　　　　　　　　　　　　　　　　　　　　　　Other neuronal activity　　　　　　　　　　　　　　　　　　　　　　　　　　　　　　　　　　　　　　　　　　　　　　　　　　　　　　　　　　　　　　　　　Complement-mediated immunity　　　　　　　　　　　　　　　　　　　　　　　　　　　　　　　　　　　　　　　　　　　　　　　　　　　　　　Receptor protein serine/threonine kinase signaling pathway　　　　　　　　　　　　　　　Extracellular matrix protein-mediated signaling　　　　　　　　　　　　　　　　　　　　　　　　　　　　　　　　　　　　　　　　　Regulation of lipid, fatty acid and steroid metabolism | 99　　　　　　　　　　　　75　　　　　　　　　　　　　44　　　　　　　　　　　　　41　　　　　　　　　　　　　29　　　　　　　　　　　　28　　　　　　　　　　　　　27　　　　　　　　　　　　　25　　　　　　　　　　　　　23　　　　　　　　　　　　　21　　　　　　　　　　　　　19　　　　　　　　　　　　18　　　　　　　　　　　　16　　　　　　　　　　　　　12　　　　　　　　　　　　10　　　　　　　　　　　　　　　7　　　　　　　　　　　　　　　　6　　　　　　　　　　　　　　　　6　　　　　　　　　　　　　　　　4 | 0.002919　　　　0.000049　　　　　0.001303　　　　　0.023365　　　　　0.005961　　　　0.003200　　　　0.001095　　　　0.003818　　　　　0.036627　　　　　0.024555　　　　　0.005227　　　　0.005356　　　　　0.010232　　　　　0.024337　　　　　0.004327　　　　　0.001722　　　　　0.002205　　　　　0.013959　　　　0.025696 |
| *Molecular Function* | | |
| Term | count | p-value |
| Cytoskeletal protein　　　　　　　　　　　　　　　　　　　　　　　　　　　　　　　　　　　　　　　　　　　　　　　　　　　　　　　　　　　　　　　　　　　　Kinase　　　　　　　　　　　　　　　　　　　　　　　　　　　　　　　　　　　　　　　　　　　　　　　　　　　　　　　　　　　　　　　　　　　　　　　　　　　　　　　　　　　　　　　　　　　　　　　　　　　　　　　Cell adhesion molecule　　　　　　　　　　　　　　　　　　　　　　　　　　　　　　　　　　　　　　　　　　　　　　　　　　　　　　　　　　　　　　　　　　　Extracellular matrix　　　　　　　　　　　　　　　　　　　　　　　　　　　　　　　　　　　　　　　　　　　　　　　　　　　　　　　　　　　　　　　　　　　　　　　　　　　　　Protein kinase　　　　　　　　　　　　　　　　　　　　　　　　　　　　　　　　　　　　　　　　　　　　　　　　　　　　　　　　　　　　　　　　　　　　　　　　　　　　　　　　　　　　　　　　Actin binding cytoskeletal protein　　　　　　　　　　　　　　　　　　　　　　　　　　　　　　　　　　　　　　　　　　　　　　　　Defense/immunity protein　　　　　　　　　　　　　　　　　　　　　　　　　　　　　　　　　　　　　　　　　　　　　　　　　　　　　　　　　　　　　　　　　　　　　　Complement component　　　　　　　　　　　　　　　　　　　　　　　　　　　　　　　　　　　　　　　　　　　　　　　　　　　　　　　　　　　　　　　　　　　　　　　　CAM family adhesion molecule　　　　　　　　　　　　　　　　　　　　　　　　　　　　　　　　　　　　　　　　　　　　　　　　　　　　　　　　　　Myelin protein | 27　　　　　　　　　　　　23　　　　　　　　　　　　　22　　　　　　　　　　　　　20　　　　　　　　　　　　　19　　　　　　　　　　　　　15　　　　　　　　　　　　14　　　　　　　　　　　　　　　7　　　　　　　　　　　　　　　　6　　　　　　　　　　　　　　　　3 | 0.031135　　　　0.033910　　　　　0.000097　　　　0.000477　　　　　0.034255　　　　　0.028632　　　　　0.043411　　　　0.000829　　　　　0.020622　　　　　0.029533 |
| *KEGG pathway* | | |
| Term | count | p-value |
| Hypertrophic cardiomyopathy (HCM)　　　　　　　　　　　　　　　　　　　　　　　　　　　　　　　　　　　　　　　　　 TGF-beta signaling pathway　　　　　　　　　　　　　　　　　　　　　　　　　　　　　　　　　　　　　　　　　　　　　　　　　　　　　　　　　　　　　　　Steroid hormone biosynthesis　　　　　　　　　　　　　　　　　　　　　　　　　　　　　　　　　　　　　　　　　　　　　　　　　　　　Complement and coagulation cascades　　　　　　　　　　　　　　　　　　　　　　　　　　　　　　　　　　　ECM-receptor interaction | 8　　　　　　　　　　　　　　　　　8　　　　　　　　　　　　　　　　　7　　　　　　　　　　　　　　　　　　　　　　　7　　　　　　　　　　　　　　　　　7 | 0.011382　　　　0.012843　　　　0.001967　　　　　0.014586　　　　0.034771 |
