# Supplementary material for: Genome-Wide DNA Methylation Profiling in Cultured Eutopic and Ectopic Endometrial Stromal Cells
Source: PLoS One. 2014 Jan 23;9(1):e83612. doi: 10.1371/journal.pone.0083612 (PMC3900404; doi:10.1371/journal.pone.0083612)
Supplement: Table S3 — Lists of statistically significant GO terms (biological process and molecular function) and KEGG pathway terms in low expressed genes in choESC compared to euESCa. (DOCX) [file pone.0083612.s005.docx]

Table S3. Low expression in choESC compred to euESCa

| *Biological Process* | | |
| --- | --- | --- |
| Term | count | p-value |
| Signal transduction　　　　　　　　　　　　　　　　　　　　　　　　　　　　　　　　　　　　　　　　　　　　　　　　　　　　　　　　Developmental processes　　　　　　　　　　　　　　　　　　　　　　　　　　　　　　　　　　　　　　　　　　　　　　　　　　　　　　　　　　　　　　　　Cell communication　　　　　　　　　　　　　　　　　　　　　　　　　　　　　　　　　　　　　　　　　　　　　　　　　　　　　　　　　　　　　　　　　　　　　　　　　　　　　　　Cell surface receptor mediated signal transduction　　　　　　　　　　　　　　　　　　　　　　　　　　　　　　　　　　　　Ectoderm development　　　　　　　　　　　　　　　　　　　　　　　　　　　　　　　　　　　　　　　　　　　　　　　　　　　　　　　　　　　　Neurogenesis　　　　　　　　　　　　　　　　　　　　　　　　　　　　　　　　　　　　　　　　　　　　　　　　　　　　　　　　　　　　　　　　　　　　　　　　　　　　　　　　　　　　　　　　　　　Cell adhesion　　　　　　　　　　　　　　　　　　　　　　　　　　　　　　　　　　　　　　　　　　　　　　　　　　　　　　　　　　　　　　　　　　　　　　　　　　　　　　　　　　　　　　　　　　　　Cell adhesion-mediated signaling　　　　　　　　　　　　　　　　　　　　　　　　　　　　　　　　　　　　　　　　　　　　　　　　　　　　　　　　　　　Cell proliferation and differentiation　　　　　　　　　　　　　　　　　　　　　　　　　　　　　　　　　　　　　　　　　　　　　　　Proteolysis　　　　　　　　　　　　　　　　　　　　　　　　　　　　　　　　　　　　　　　　　　　　　　　　　　　　　　　　　　　　　　　　　　　　　　　　　　　　　　　　　　　　　　　Neuronal activities　　　　　　　　　　　　　　　　　　　　　　　　　　　　　　　　　　　　　　　　　　　　　　　　　　　　　　　　　　　　　　　　　　　　　　　　　Mesoderm development　　　　　　　　　　　　　　　　　　　　　　　　　　　　　　　　　　　　　　　　　　　　　　　　　　　　　　　　　　　　　　　　　　Receptor protein tyrosine kinase signaling pathway　　　　　　　　　　　　　　　　　　　　　　　　　　　　　　　Synaptic transmission　　　　　　　　　　　　　　　　　　　　　　　　　　　　　　　　　　　　　　　　　　　　　　　　　　　　　　　　　　　　　　　　　　　　　　Skeletal development　　　　　　　　　　　　　　　　　　　　　　　　　　　　　　　　　　　　　　　　　　　　　　　　　　　　　　　　　　　　　　　Angiogenesis　　　　　　　　　　　　　　　　　　　　　　　　　　　　　　　　　　　　　　　　　　　　　　　　　　　　　　　　　　　　　　　　　　　　　　　　　　　　　　　　　　　　　　　　Blood clotting | 125　　　　　　　　　　98　　　　　　　　　　　　　　　　　　　61　　　　　　　　　　　　　　　59　　　　　　　　　　　　　　　　　　　　49　　　　　　　　　　　　　　　　　47　　　　　　　　　　　　　　　　39　　　　　　　　　　　　　　　　33　　　　　　　　　　　　　　　　　　　　　　31　　　　　　　　　　　　　　　　　　30　　　　　　　　　　　　　　　　　　　　23　　　　　　　　　　　　　　　　　　22　　　　　　　　　　　　　　　　19　　　　　　　　　　　　13　　　　　　　　　　　　　　　　　　　　　9　　　　　　　　　　　　　　　　　　8　　　　　　　　　　　　　　　　6 | <0.000001　<0.000001　<0.000001　　　0.000049　　<0.000001　<0.000001　<0.000001　<0.000001　　0.032489　　　　0.017750　　　　0.004680　　　　0.007075　　　<0.000001　　0.015687　　　　0.005622　　　　0.000143　　　　0.044571 |
| *Molecular Function* | | |
| Term | count | p-value |
| Receptor　　　　　　　　　　　　　　　　　　　　　　　　　　　　　　　　　　　　　　　　　　　　　　　　　　　　　　　　　　　　　　　　　　　　　　　　　　　　　　　　　Signaling molecule　　　　　　　　　　　　　　　　　　　　　　　　　　　　　　　　　　　　　　　　　　　　　　　　　　　　　　　　　　　　　　　　　　　　　　　　　　　　Cell adhesion molecule　　　　　　　　　　　　　　　　　　　　　　　　　　　　　　　　　　　　　　　　　　　　　　　　　　　　　　　　　　　Extracellular matrix　　　　　　　　　　　　　　　　　　　　　　　　　　　　　　　　　　　　　　　　　　　　　　　　　　　　　　　　　　　　　　　　　　　　　　　　　　Protease　　　　　　　　　　　　　　　　　　　　　　　　　　　　　　　　　　　　　　　　　　　　　　　　　　　　　　　　　　　　　　　　　　　　　　　　　　　　　　　　　　　　　　　　　　　Cadherin　　　　　　　　　　　　　　　　　　　　　　　　　　　　　　　　　　　　　　　　　　　　　　　　　　　　　　　　　　　　　　　　　　　　　　　　　　　　　　　Metalloprotease　　　　　　　　　　　　　　　　　　　　　　　　　　　　　　　　　　　　　　　　　　　　　　　　　　　　　　　　　　　　　　　　　　　　　　　　　　　　　　　　　　　Other signaling molecule　　　　　　　　　　　　　　　　　　　　　　　　　　　　　　　　　　　　　　　　　　　　　　　　　　　　　　　　　　　　　Homeobox transcription factor　　　　　　　　　　　　　　　　　　　　　　　　　　　　　　　　　　　　　　　　　　　　　　　　　　　　　　　　　　Membrane-bound signaling molecule　　　　　　　　　　　　　　　　　　　　　　　　　　　　　　　　　　　　　　　　　　　　　　　　　　Other extracellular matrix　　　　　　　　　　　　　　　　　　　　　　　　　　　　　　　　　　　　　　　　　　　　　　　　　　　　　　　　　　　　　　　　　Tyrosine protein kinase receptor　　　　　　　　　　　　　　　　　　　　　　　　　　　　　　　　　　　　　　　　　　　　　　　　　　Extracellular matrix glycoprotein　　　　　　　　　　　　　　　　　　　　　　　　　　　　　　　　　　　　　　　　　　　　　　　　　　　　　　　　　　　　　Protein kinase receptor　　　　　　　　　　　　　　　　　　　　　　　　　　　　　　　　　　　　　　　　　　　　　　　　　　　　　　　　　　　　　　Extracellular matrix structural protein | 51　　　　　　　　　　　　34　　　　　　　　　　　　　30　　　　　　　　　　　　　29　　　　　　　　　　　　　　　　22　　　　　　　　　　　　　　　　　　　17　　　　　　　　　　　　　　　　　　14　　　　　　　　　　　　　　　　14　　　　　　　　　　　　　　　　　　13　　　　　　　　　　　　　　　　　　　10　　　　　　　　　　　　　　　　　　　　　9　　　　　　　　　　　　　　　　　7　　　　　　　　　　　　　　　　　7　　　　　　　　　　　　　　　　　　7　　　　　　　　　　　　　　　　　6 | 0.000605　　　　0.000083　　　<0.000001　<0.000001　　0.004456　　<0.000001　　0.000030　　　　0.002508　　　　0.003964　　　　0.001150　　　　0.000001　　　　0.004917　　　　　0.017233　　　　　0.021684　　　　0.034068 |
| *KEGG pathway* | | |
| Term | count | p-value |
| Focal adhesion　　　　　　　　　　　　　　　　　　　　　　　　　　　　　　　　　　　　　　　　　　　　　　　　　　　　　　　　　　　　　　　　　　　　Neuroactive ligand-receptor interaction　　　　　　　　　　　　　　　　　　　　　　　　　　　　　　　　　　Pathways in cancer  Calcium signaling pathway　　　　　　　　　　　　　　　　　　　　　　　　　　　　　　　　　　　　　　　　　Arrhythmogenic right ventricular cardiomyopathy (ARVC)　　 ECM-receptor interaction　　　　　　　　　　　　　　　　　　　　　　　　　　　　　　　　　　　　　　　　　　　　　　　　　　　Complement and coagulation cascades　　　　　　　　　　　　　　　　　　　　　　　　　　　　　　　　　　　　　　　　　Small cell lung cancer　　　　　　　　　　　　　　　　　　　　　　　　　　　　　　　　　　　　　　　　　　　　　　　　　　　　　　　　　　　　　　　　　　　　　　　Type II diabetes mellitus | 18　　　　　　　　　　　　17　　　　　　　　　　　　16　　　　　　　　　　　　　10　　　　　　　　　　　　　　　7　　　　　　　　　　　　　　　　　7　　　　　　　　　　　　　　　　　6　　　　　　　　　　　　　　　　　6　　　　　　　　　　　　　　　　　5 | 0.000003　　　　0.000218　　　　　0.007722　　　　　0.019411　　　　　0.007696　　　　　0.012340　　　　0.020649　　　　　0.043343　　　　　0.022193 |
