# Supplementary material for: Genome-Wide DNA Methylation Profiling in Cultured Eutopic and Ectopic Endometrial Stromal Cells
Source: PLoS One. 2014 Jan 23;9(1):e83612. doi: 10.1371/journal.pone.0083612 (PMC3900404; doi:10.1371/journal.pone.0083612)
Supplement: Table S4 — Lists of genes that have significant hypermethylated CpGs with significant low mRNA expressions in choESC compared to euESCa. The significant GO terms were shown below the gene lists. (DOCX) [file pone.0083612.s006.docx]

Table S4. Hypermethylation - Low expression

|  |  | Beta value | | | | | |  |
| --- | --- | --- | --- | --- | --- | --- | --- | --- |
| TargetID | SYMBOL | euESCa Average | | choESC Average | | ⊿ | | Fold change |
| cg00075967　cg24691453　cg23207527　cg15626350　cg16466334　cg26894575　cg11787522　cg00217795　cg03918304　cg21831174　cg19612574　cg11946165　cg16478145　cg06259570　cg05125838　cg17083925　cg02727423　cg16708012　cg20373326　cg09396217　cg08477744　cg06490988　cg19264571　cg19280776 | STRA6　　　　　　S100A4　　　　　RBM24　　　　　　　　　ESR1　　　　　　　　　　　MMP3　　　　　　　　S100A4　　　　　　STRA6　　　　　　　　　　DIO2　　　　　　　HOXD10　　　　MASP1　　　　　MAPK8　　　　　　　　CTSK　　　　　　　　　　　　　TFPI　　　　　　　　　　MMP27　　　　　　　　UCN2　　　　　　　　　　TGM2　　　　　　　　　　　EML1　　　　TMEM119　HSD17B2　　ANGPT1　　　MFAP2　　　　　　GATA2　　　　APCDD1　　　　　　PAG1 | 0.075　　　　　　　　　　　　　0.274　　　　　　　　　　　　　　0.263 0.065 0.155 0.041 0.077 0.080 0.051 0.169 0.248 0.176 0.250 0.344 0.448 0.070 0.368 0.109 0.486 0.279 0.572 0.030 0.175 0.059 | | 0.808 0.888 0.863 0.649 0.691 0.575 0.576 0.548 0.498 0.600 0.630 0.552 0.616 0.660 0.729 0.337 0.629 0.366 0.734 0.522 0.791 0.243 0.386 0.261 | | 0.732 0.614 0.600 0.585 0.536 0.535 0.499 0.468 0.446 0.431 0.383 0.376 0.366 0.316 0.281 0.267 0.261 0.256 0.248 0.243 0.219 0.213 0.210 0.203 | | -13.22 -2.20 -2.21 -4.47 -71.72 -2.20 -13.22 -23.89 -18.15 -2.56 -2.66 -2.09 -2.21 -5.09 -3.01 -4.14 -2.38 -3.11 -16.94 -6.12 -6.51 -4.16 -5.19 -2.93 |
| *Biological Process* | | |  | | | | | |
| Term | | | Count | | p-value | | Genes | |
| Proteolysis | | | 5 | | 0.01228 | | CTSK, MASP1, MMP27, TFPI, MMP3 | |
| *Molecular Function* | | | | | | | | |
| Term | | | Count | | p-value | | Genes | |
| Protease Other extracellular matrix | | | 4 2 | | 0.019076 0.0405075 | | CTSK, MASP1, MMP27, MMP3 MMP27, MMP3 | |
