# Supplementary material for: Genome-Wide DNA Methylation Profiling in Cultured Eutopic and Ectopic Endometrial Stromal Cells
Source: PLoS One. 2014 Jan 23;9(1):e83612. doi: 10.1371/journal.pone.0083612 (PMC3900404; doi:10.1371/journal.pone.0083612)
Supplement: Table S5 — Lists of genes that have significant hypomethylated CpGs with significant high mRNA expressions in choESC compared to euESCa. The significant GO and KEGG pathway terms were shown below the gene lists. (DOCX) [file pone.0083612.s007.docx]

Table S5. Hypomethylation - High expression

|  |  | Beta value | | | | | |  |
| --- | --- | --- | --- | --- | --- | --- | --- | --- |
| TargetID | SYMBOL | euESCa Average | | choESC Average | | ⊿ | | Fold change |
| cg24215443 cg24754277 cg13718960 cg10771262 cg16363586 cg16666160 cg23732024 cg24315815 cg05958352 cg22627427 cg00691625 cg12943082 cg08124030 cg08399444 cg15514848 cg04237003 cg24928687 cg21932814 cg26928972 cg09793866 cg19780563 cg03430998 cg17088290 cg02554564 cg16176600 cg15779716 cg05556717 cg08493463 cg15746719 cg26020513 cg24579667 cg21750589 cg18939260 cg19172575 cg00446235 cg01770232 cg21073927 cg07327468 cg18123948 | TCF21 DAPK1 RNASE1 TCF21 BST2 NR5A1 LY96 PLSCR4 RNASE1 C11orf9 NR5A1 CCL26 TM4SF1 GSG1 FMO1 C11orf9 EPHX1 CSTA CSTA STAR EXOC6 TLR1 AMHR2 NTF3 FRK CDCP1 CCL26 ENPP6 DAPK1 GATA4 GPD2 ZNF323 MTSS1 GATA4 F11R IL6 GATA4 EYA4 GATA4 | 0.827 0.828 0.852 0.818 0.830 0.975 0.714 0.743 0.853 0.882 0.673 0.726 0.838 0.869 0.654 0.648 0.844 0.635 0.522 0.713 0.517 0.594 0.838 0.778 0.624 0.897 0.555 0.639 0.345 0.520 0.389 0.606 0.866 0.381 0.286 0.467 0.233 0.603 0.260 | | 0.081 0.089 0.122 0.116 0.130 0.325 0.080 0.114 0.243 0.339 0.132 0.204 0.319 0.366 0.153 0.157 0.406 0.198 0.127 0.322 0.144 0.230 0.480 0.420 0.269 0.543 0.223 0.316 0.037 0.213 0.100 0.317 0.577 0.128 0.045 0.248 0.027 0.399 0.058 | | -0.746 -0.738 -0.730 -0.702 -0.699 -0.650 -0.634 -0.629 -0.611 -0.543 -0.541 -0.522 -0.520 -0.503 -0.501 -0.491 -0.439 -0.437 -0.396 -0.391 -0.373 -0.364 -0.359 -0.358 -0.355 -0.354 -0.332 -0.323 -0.308 -0.308 -0.289 -0.289 -0.288 -0.253 -0.241 -0.219 -0.206 -0.204 -0.202 | | 14.094 10.724 3.123 14.094 11.194 3.012 3.329 5.097 3.123 5.674 3.012 8.179 3.147 6.494 3.954 5.674 2.357 5.163 5.163 53.104 2.380 2.765 4.608 2.279 2.111 3.614 8.179 2.589 10.724 5.832 2.071 2.511 3.640 5.832 2.084 4.730 5.832 4.235 5.832 |
| *Biological Process* | | |  | | | | | |
| Term | | | Count | | p-value | | Genes | |
| Cytokine and chemokine mediated signaling pathway Developmental processes | | | 4 9 | | 0.0072 0.0151 | | AMHR2, IL6, TLR1, CCL26 AMHR2, FRK, EYA4, MTSS1, STAR, NTF3, GATA4, TLR1, NR5A1 | |
| *Molecular Function* | | | | | | | | |
| Term | | | Count | | p-value | | Genes | |
| Toll-like receptor signaling pathway | | | 3 | | 0.0191 | | IL6, LY96, TLR1 | |
