# Supplementary material for: Genome-Wide DNA Methylation Profiling in Cultured Eutopic and Ectopic Endometrial Stromal Cells
Source: PLoS One. 2014 Jan 23;9(1):e83612. doi: 10.1371/journal.pone.0083612 (PMC3900404; doi:10.1371/journal.pone.0083612)
Supplement: Table S6 — Lists of genes that have significant hypo-methylated CpGs with significant low mRNA expressions in choESC compared to euESCa. The significant GO terms were shown below the gene lists. (DOCX) [file pone.0083612.s008.docx]

Table S6. Hypomethylation - Low expression

|  |  | Beta value | | | | | |  |
| --- | --- | --- | --- | --- | --- | --- | --- | --- |
| TargetID | SYMBOL | euESCa Average | | choESC Average | | ⊿ | | Fold change |
| cg26069745 cg09871315 cg09017174 cg11809085 cg22549408 cg05996042 cg01946401 cg06958537 cg09313705 cg22199118 | HOXA2 HOXA2 SLC1A2 TNFSF15 PMAIP1 RUNX2 RUNX2 ETS2 HOXB2 C8orf34 | 0.906 0.793 0.649 0.750 0.786 0.657 0.774 0.421 0.786 0.613 | | 0.300 0.221 0.139 0.303 0.365 0.291 0.418 0.072 0.445 0.341 | | -0.606 -0.572 -0.510 -0.447 -0.421 -0.366 -0.356 -0.349 -0.341 -0.272 | | -3.09 -3.09 -3.20 -9.92 -2.95 -2.27 -2.27 -2.89 -2.87 -2.20 |
| *Biological Process* | | |  | | | | | |
| Term | | | Count | | p-value | | Genes | |
| Developmental processes  Oncogenesis Segment specification | | | 5  3 2 | | 0.007038  0.0142794 0.0444697 | | HOXA2, HOXB2, ETS2, TNFSF15, RUNX2 ETS2, PMAIP1, RUNX2 HOXA2, HOXB2 | |
| *Molecular Function* | | | | | | | | |
| Term | | | Count | | p-value | | Genes | |
| Transcription factor | | | 4 | | 0.0232003 | | HOXA2, HOXB2, ETS2, RUNX2 | |
