# Supplementary material for: Genome-Wide DNA Methylation Profiling in Cultured Eutopic and Ectopic Endometrial Stromal Cells
Source: PLoS One. 2014 Jan 23;9(1):e83612. doi: 10.1371/journal.pone.0083612 (PMC3900404; doi:10.1371/journal.pone.0083612)
Supplement: Table S7 — Lists of genes that have significant hypermethylated CpGs with significant high mRNA expressions in choESC compared to euESCa. (DOCX) [file pone.0083612.s009.docx]

Table S7. Hypermethylation - High expression

|  |  | Beta value | | |  |
| --- | --- | --- | --- | --- | --- |
| TargetID | SYMBOL | euESCa Average | choESC Average | ⊿ | Fold change |
| cg23801057 cg00430287 cg06020352 cg13823701 cg10876928 cg01658815 cg10559803 cg07732644 cg22074467 cg05593325 cg02228185 | P2RX7 FRK IRF8 TNXB SDC4 ABLIM1 RALGPS2 ASPA AKR1C2 GALNT12 ASPA | 0.195 0.360 0.189 0.129 0.090 0.124 0.309 0.489 0.294 0.059 0.074 | 0.719 0.780 0.606 0.471 0.432 0.407 0.585 0.729 0.527 0.287 0.302 | 0.525 0.419 0.416 0.343 0.342 0.282 0.276 0.240 0.233 0.228 0.228 | 3.09 2.11 2.34 2.68 2.95 2.43 2.42 2.13 2.99 3.96 2.13 |
